# Chicken embryo lethality assay for determining the lethal dose, tissue distribution and pathogenicity of clinical *Enterococcus cecorum* isolates from poultry

**DOI:** 10.1038/s41598-022-14900-9

**Published:** 2022-06-23

**Authors:** Beata Dolka, Michał Czopowicz, Izabella Dolka, Piotr Szeleszczuk

**Affiliations:** 1 Department of Pathology and Veterinary Diagnostics, Institute of Veterinary Medicine, Warsaw University of Life Sciences – SGGW, Nowoursynowska 159c St., 02-776 Warsaw, Poland; 2 Division of Veterinary Epidemiology and Economics, Institute of Veterinary Medicine, Warsaw University of Life Sciences – SGGW, Nowoursynowska 159c St., 02-776 Warsaw, Poland

**Keywords:** Microbiology, Bacteria, Infectious-disease diagnostics, Pathogens

## Abstract

*Enterococcus cecorum* is a well-known component of the normal poultry intestinal microbiota and an important bacterial pathogen. Infections caused by *E. cecorum* have negative effects on the poultry production worldwide. In this study we used the SPF-chicken embryo lethality assay (ELA) to assess the pathogenic potential of *E. cecorum*. A total of 23 isolates were used: 19 clinical isolates from field outbreaks in different poultry groups (CB – broiler chickens, BB – broiler breeders, CL – layers, T– turkeys, W – waterfowl) and 4 commensal isolates. The cumulative mortality caused by all clinical isolates was higher (53.4%) than that of the commensals (38.9%). The highest mortality was induced by CB isolates (68.9%), followed by CL (60.4%), all chicken isolates (59.2%; CB, BB, CL), BB (45.8%), T (41.7%), non-chicken isolates (40.7%; T, W), and W isolates (39.8%). Most of the embryos that died, did die on the 1st day post-infection (dpi), except those infected with CB, CL (on 2 dpi). The median lethal dose (LD_50_) of *E. cecorum* ranged from 6.07 × 10^2^ cfu/ml (CB isolates) and 1.42 × 10^4^ cfu/ml (all clinical isolates) to 4.8 × 10^5^ cfu/ml (commensal isolates). This study provides the first evidence of a wide tissue distribution and multiplication of *E. cecorum* in embryos. Dead embryos showed scattered petechiae, hemorrhages, aggregates of bacteria in blood vessels, multiple organ necrosis, and encephalomalacia. Our data indicate that surviving embryos were able to elicit innate immune response to infection. On the other hand, reisolation of viable bacteria from surviving embryos may suggest that *E. cecorum* could evade or resist immune mechanisms in order to persist in organs. Furthermore, body mass of surviving embryos was affected by the strain type, not the dose (bacterial concentration) used, and was lower for the infection with clinical strains. The results indicated the highest pathogenicity of clinical *E. cecorum* isolates from CB and CL flocks.

## Introduction

*Enterococcus cecorum* is a facultatively anaerobic, Gram-positive, catalase-negative and pyroglutamic acid arylamidase (PYRase)-negative bacterium^1,2^. During the last two decades, *E. cecorum* gained recognition in the field as an emerging pathogen responsible for significant losses in the poultry industry. The morbidity and mortality can vary across countries and flocks. Overall 25–35% of the broiler flock can be affected, with 5–15% mortality and nearly 10% culling rate. To date, the economic costs of *E. cecorum* infections have not been calculated and specified^3–10^. The particular predisposition to infection of meat-type chickens and the sensitivity of other poultry species to infection have been confirmed^2,11–13^. Outbreaks of *E. cecorum* infection have been reported worldwide in broiler chickens (CB), broiler breeders (BB), laying hens (CL), waterfowl (W), turkeys (T), and racing pigeons^2–4,7,10,14–16^. Studies have shown that the disease occurs in different age groups depending on the type of poultry. Some differences have also been demonstrated in terms of the clinical manifestation of infection and frequency of *E. cecorum* isolation from the affected tissue samples depending on the poultry group, which may suggest differences in pathogenesis^12^. Moreover, some differences have been noted with regard to the type of lesions depending on the poultry group, even after experimental infection^11,12^. *E. cecorum* has been implicated in various pathological conditions including vertebral osteomyelitis, femoral head necrosis, fibrinous pericarditis, fibrinous hepatitis, fibrinous pneumonia, fibrinous ovaritis, hydropericardium, and septicemia^3,4,6,8,12,14^. Joint, heart and liver lesions have been observed in all poultry groups, but spinal lesions have been reported only in broiler chickens and broiler breeder chickens^12^. The worldwide increase in the disease outbreaks may suggest the conversion of specific strains from commensal to pathogenic and the emergence of strains with increased virulence potential^5,6,17^. The roles of specific concurrent infections, unspecified (host-related and environmental) predisposing factors, and changes in genetics have also been discussed^3,4,17,18^. Our previous research demonstrated genetic heterogeneity among clinical *E. cecorum* strains derived from poultry^12^. Strains responsible for the recent emergence of associated enteroccocal diseases showed high levels of antimicrobial resistance and virulence determinants^17,19^. However, the prevalence of virulence determinants among the European pathogenic strains was relatively low^12,13^. The differences in genomic features, phenotypic properties and colonization abilities between commensal and pathogenic isolates may indicate their divergent evolution^9,13,19,20^.

The purpose of this study was to evaluate the virulence of clinical *E. cecorum* isolates from different poultry groups by using experimental infection of chicken embryos. Even though many reports on the use of chicken embryo lethality assay (ELA) in assessing the pathogenic potential of bacterial pathogens have been published^21–24^, only three referred to *E. cecorum*^13,25,26^*,* while most focused on *E. faecalis* or *E. faecium*^22,27–30^. Therefore, we aimed to determine the median lethal dose (LD_50_) of *E. cecorum* from different poultry groups and to identify pathomorphological lesions in embryos, preferential tissue distribution and bacterial load.

## Methods

### Bacterial strains

A total of 23 *E. cecorum* isolates were used in this study: 19 clinical (pathogenic) strains previously isolated from independent field infection cases in poultry between 2011 and 2017 in Poland (Supplementary Table 1), 4 commensal strains from the ceca of healthy chickens, including 1 type strain (ATCC 43,198) from chicken ceca. Clinical field isolates represented different poultry species, production groups, flocks (5 chicken broilers – CB, 4 broiler breeders – BB, 4 layers – CL, 3 turkeys – T, 3 waterfowl – W), years, geographic locations, and clones (based on the PFGE pulsotypes). Isolates were retrieved from tissues demonstrating pathological lesions and characterized in our previous study^12^.

### Inoculum preparation

All bacterial cultures were incubated overnight microaerobically at 37 °C on Columbia agar plates with sheep blood (CA, Graso, Poland). The stock solutions were prepared by dissolving colonies of each *E. cecorum* isolate (n = 23) in saline to match the turbidity of 1.0 McFarland standard (DEN-1, Biosan, Riga, Latvia), which corresponds to approximately 3.4 × 10^8^ colony-forming units/ml (cfu/ml). The bacterial concentration was confirmed for each isolate using the standard enumeration method, by spreading 100 μl of dilutions on triplicate Enterococcosel agar plates (Graso, Poland). The stock solution and eight dilutions (up to 10^–8^) of each isolate were used for inoculation.

### Experimental design

The ELA was performed on fertile specific pathogen–free (SPF) chicken eggs (VALO BioMedia GmbH, Germany) according to the procedure adopted from the literature^25,31^. Before inoculation, the eggs were candled to determine embryo viability, then the air cell, large blood vessels, and embryo location were marked. The eggshell injection site (over the air cell) was disinfected with iodine and penetrated with a sterile needle. Groups of 11-day-old embryonated SPF eggs were inoculated into the chorioallantoic sac (CAS) with 0.1 ml of each bacterial solution (4 eggs were used per each dose). In addition to the infected embryos, there was a control group which consisted of 4 embryos inoculated with sterile saline and another one non-inoculated. Every inoculation was done with a sterile 1 ml syringe (Medical – Łomża, Poland) and a 23G needle (0.6 × 25 mm). After inoculation, the opening was sealed with a small piece of tape. All eggs were then incubated in the same incubator (Heka Incubator, Przewoz, Poland) at 37 °C, 55% humidity, without turning, for 7 days with daily candling to determine the embryo mortality rate (EMR). Embryos that survived until the 7th day post-infection (dpi), i.e. until the 18th day of incubation (di), were chilled at 4 °C for one hour.

### Median lethal dose (LD_50_)

The LD_50_ was calculated using the Reed and Muench method based on the cumulative number of dead embryos and surviving embryos at each dilution^32,33^. The cumulative mortality caused by all *E. cecorum* strains of each poultry group were used for calculations.

### Pathomorphological lesions

Dead and survived embryos were necropsied for gross lesions. Fresh tissue samples were collected from embryos on the day of necropsy and fixed in 10% buffered formalin. The tissue samples were submitted to the Division of Animal Pathology, Department of Pathology and Veterinary Diagnostics, Institute of Veterinary Medicine at the Warsaw University of Life Sciences—SGGW, Poland, for histopathological examination. The samples were routinely processed: dehydrated in increasing gradients of ethyl alcohol, embedded in paraffin and cut into 4 µm-thick sections using a microtome. Subsequently, paraffin sections were stained with hematoxylin and eosin (HE). The samples were analysed under the BX41 light microscope (Olympus, Japan).

### Reisolation of *E. cecorum*

The specific organ samples were tested separately for each *E. cecorum* isolate. Yolk sac, heart, and gizzard (muscular stomach) samples from dead (on 13 di, 2 dpi) and surviving (on 18 di, 7 dpi) embryos that were inoculated with the highest bacterial concentration (approx. 3.4 × 10^7^ cfu/egg) were used for reisolation of *E. cecorum*. Tissue samples were cut using a sterile scalpel blade and homogenized with sterile saline on a vortex mixer until complete disruption and obtaining homogeneous tissue suspensions. The plate count method (on Enterococcosel agar, Graso, Poland) was used to determine the total number of bacteria found in the organs mentioned. The total number of bacteria was expressed as cfu/g of organ (yolk sac, heart, gizzard) and cfu/heart (total heart weight). Identification of reisolated *E. cecorum* was confirmed by colony morphology on CA plates, catalase test or PCR with species-specific primers^34^.

### Body mass of embryos

Embryos that survived infection until the last day of the experiment (7 dpi, 18 di) were weighed using an Ohaus® PA214CM/1 scale (max: 210 g, min: 0.01 g, e = 0.001 g, d = 0.0001 g, Ohaus®, USA).

### Statistical analysis

Numerical variables were presented as an arithmetic mean and standard deviation (SD), or a median and interquartile range (IQR), depending on the shape of their distribution. Range was given in all cases. Numerical variables were compared between unpaired groups with the Mann–Whitney U test, and between paired groups using the Friedman or Wilcoxon signed rank test, with the Bonferroni correction in the case of multiple comparisons. Categorical variables were given as counts and percentages, which were then compared between groups using the Pearson χ^2^ test. The ninety five per cent confidence intervals (95% CI) for proportions were calculated according to the Wilson score method. When more than two groups were compared, the χ^2^ test was considered an omnibus test. Therefore, when it yielded a significant result, a post-hoc analysis was performed according to the procedure described by Markowski and Markowski^35^. Briefly, the group with the largest average contribution to the χ^2^total was identified and removed from the contingency table, and the χ^2^ test was performed again. The procedure was repeated until the χ^2^ test yielded an non-significant result.

Embryo survival probability was analysed using the Kaplan–Meyer plots and compared between groups with the log-rank test. The influence of the dilution (dose) and the type or groups of strains on mortality was investigated using the multivariable logistic regression model, and their role was presented as odds ratio (OR). The influence of the dilution and the type or groups of strains on the body mass of embryos (Y_BM_) was investigated using the general linear model (GLM) with dilution and clinical type of strains (X_clinical_) (model 1) or dilution and groups of strains (X_CB_, X_BB_, X_CL_, X_T_, X_W_) (model 2) fitted in the GLM as fixed factors, and commensal strains serving as a reference category. GLMs were expressed with the equation:

$$\begin{aligned} & {\text{Y}}_{{{\text{BM}}}} = {\text{B}}_{0} + {\text{B}}_{{{\text{dilution}}}} \times {\text{X}}_{{{\text{dilution}}}} + {\text{B}}_{{{\text{clinical}}}} \times {\text{X}}_{{{\text{clinical}}}} \\ & {\text{Y}}_{{{\text{BM}}}} = {\text{B}}_{0} + {\text{B}}_{{{\text{dilution}}}} \times {\text{X}}_{{{\text{dilution}}}} + {\text{B}}_{{{\text{CB}}}} \times {\text{X}}_{{{\text{CB}}}} + {\text{B}}_{{{\text{BB}}}} \times {\text{X}}_{{{\text{BB}}}} + {\text{B}}_{{{\text{CL}}}} \times {\text{X}}_{{{\text{CL}}}} + {\text{B}}_{{\text{T}}} \times {\text{X}}_{{\text{T}}} + {\text{B}}_{{\text{W}}} \times {\text{X}}_{{\text{W}}} \\ \end{aligned}$$

B_0_ was the intercept, and B with a relevant subscript stood for the coefficient of regression of a given explanatory variable.

All statistical tests were two-sided. The significance level (α) was set at 0.05. Statistical analysis was performed in TIBCO Statistica 13.3 (TIBCO Software Inc., Palo Alto, CA, USA). GLMs were developed in IBM SPSS Statistics 26 (IBM Corporation, Armonk, NY).

### Ethics approval

The Approval of Animal Ethics Commission was not required for the presented work according to the Polish law (the Act on the Protection of Animals Used for Scientific or Educational Purposes of 15 January 2015, published in the Journal of Laws of 2015 as item 266) and the European Union regulations (Directive 2010/63/EU of the European Parliament and of the Council of 22 September 2010 on the protection of animals used for scientific purposes). The experiment on SPF chicken embryos was completed 3 days prior to hatching, on developmental day 18 at the latest. All methods were carried out in accordance with the relevant guidelines and regulations. The study was carried out in compliance with the ARRIVE guidelines.

## Results

### Embryo mortality rate

EMRs for each dose of clinical and commensal *E. cecorum* strains are shown in Table 1. Analysis of the effect of strain type and dose on EMR showed that mortality decreased along with decreasing infective dose, both in clinical and commensal *E. cecorum* strains (*p < *0.001). Infection with clinical strains doubled mortality at all doses compared with infection with commensal strains (*p = *0.001, OR 1.90, 95% CI: 1.29, 2.79). Dose–response analysis showed that infection with strains belonging to groups CB and CL significantly increased mortality, roughly 2–4 fold, compared with commensal strains (CB *p < *0.001, OR 3.47, 95% CI: 2.37, 5.08; and CL *p < *0.001, OR 2.31, 95% CI: 1.55, 3.44). Infection with strains from the remaining groups was not significantly linked to mortality compared with commensal strains (BB *p = *0.209, OR 1.30, 95% CI: 0.86, 1.96; T *p = *0.658, OR 1.12, 95% CI: 0.69, 1.80; and W *p = *0.876, OR 1.04,95% CI: 0.61, 1.78).

**Table 1:** Embryo mortality with decreasing dose of clinical (CB, BB, CL, T, W) and commensal *Enterococcus cecorum* strains from poultry. Results are presented as % mortality rate (with 95% CI) and the number of deaths / total embryos.

Inoculum (cfu/ml)	*E. cecorum* strains derived from different poultry groups					
	CB (n = 5)	BB (n = 4)	CL (n = 4)	T (n = 3)	W (n = 3)	Commensal (n = 4)
3.4 × 10^8^	90 (70, 97) 18/20	69 (44, 86) 11/16	69 (44, 86) 11/16	50 (25, 75) 6/12	50 (25, 75) 6/12	68.8 (44, 86) 11/16
3.4 × 10^7^	85 (64, 95) 17/20	50 (28, 72) 8/16	75 (51, 90) 12/16	42 (19, 68) 5/12	50 (25, 75) 6/12	63 (39, 82) 10/16
3.4 × 10^6^	85 (64, 95) 17/20	56 (33, 77) 9/16	69 (44, 86) 11/16	58 (32, 81) 7/12	50 (25, 75) 6/12	50 (28, 72) 8/16
3.4 × 10^5^	80 (58, 92) 16/20	50 (28, 72) 8/16	88 (64, 97) 14/16	58 (32, 81) 7/12	42 (19, 68) 5/12	31 (14, 56) 5/16
3.4 × 10^4^	85 (64, 95) 17/20	56 (33, 77) 9/16	38 (19, 61) 6/16	42 (19, 68) 5/12	50 (25, 75) 6/12	38 (19, 61) 6/16
3.4 × 10^3^	80 (58, 92) 16/20	38 (19, 61) 6/16	69 (44, 86) 11/16	42 (19, 68) 5/12	33 (14, 61) 4/12	31 (14, 56) 5/16
3.4 × 10^2^	50 (30, 70) 10/20	56 (33, 77) 9/16	88 (64, 97) 14/16	33 (14, 61) 4/12	33 (14, 61) 4/12	31 (14, 56) 5/16
3.4 × 10	50 (30, 70) 10/20	31 (14, 56) 5/16	31 (14, 56) 5/16	25 (9, 53) 3/12	33 (14, 61) 4/12	19 (7, 43) 3/16
3.4	15 (5, 36) 3/20	6 (1, 28) 1/16	19 (6.6, 43) 3/16	25 (9, 53) 3/12	17 (5, 45) 2/12	19 (7, 43) 3/16

**Figure 1:**
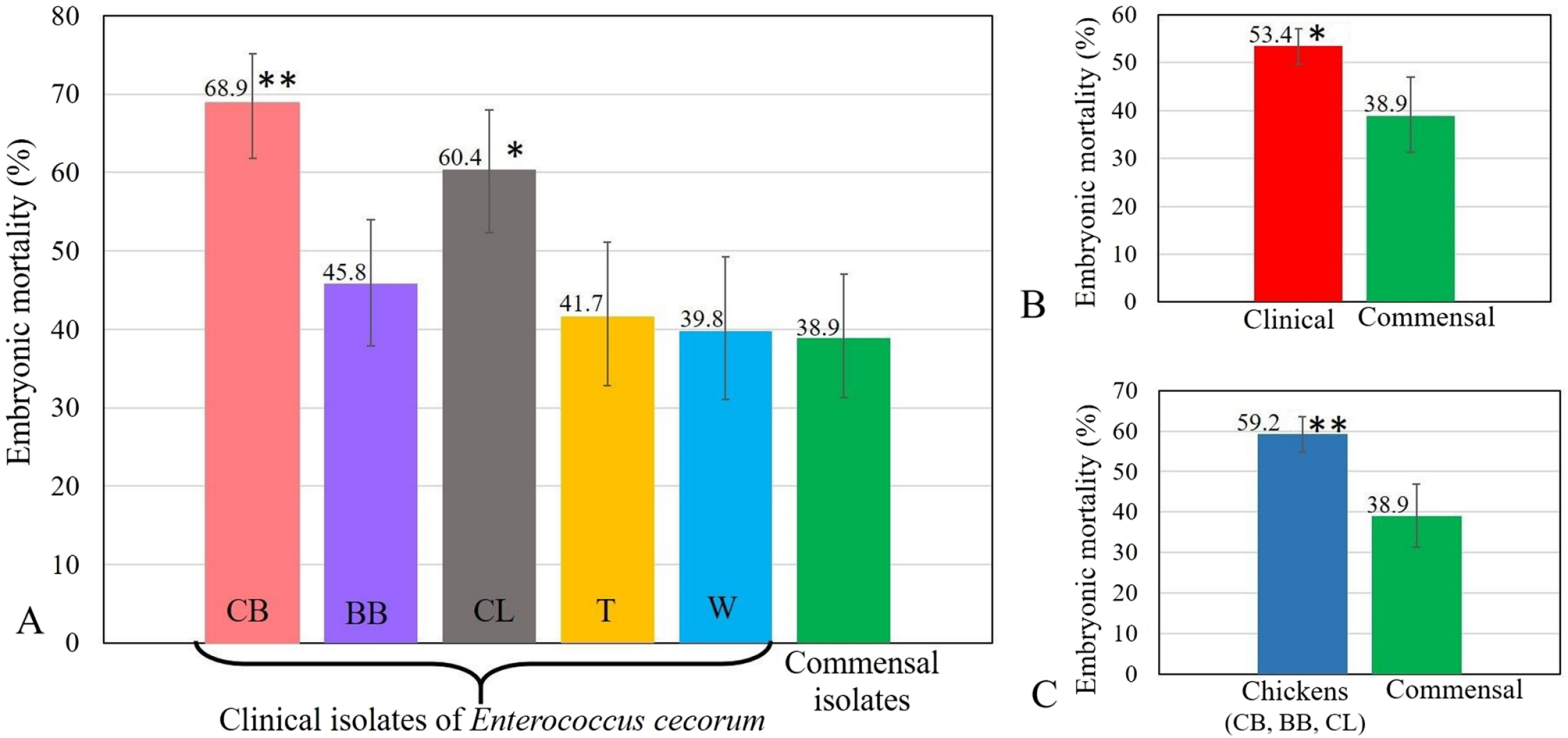
Cumulative embryo mortality rate (%) following infection with all doses of **(A)** clinical CB (n = 5), BB (n = 4), CL (n = 4), T (n = 3), W (n = 3) and commensal (n = 4) *Enterococcus cecorum* strains **(B)** all clinical (n = 19) and commensal (n = 4) *E. cecorum* strains **(C)** all clinical chicken isolates (n = 13; CB, BB, CL) and commensal *E. cecorum*. **p < 0.001, *p < 0.05.

The cumulative EMR determined for all *E. cecorum* strains at all doses is presented in Fig. 1A–C, Table 2, Supplementary Table 2, and Supplementary Table 3. The cumulative EMR caused by all clinical strains was estimated at 53.4% with 365 deaths of 684 embryos (95% CI: 49.6, 57.1) and was significantly higher (*p = *0.002) than mortality caused by all commensal strains: 38.9% (95% CI: 31.3, 47.0; 56/144) (Fig. 1B). The cumulative EMR was significantly higher in embryos inoculated with *E. cecorum* strains from CB flocks: 68.9% (95% CI: 61.8, 75.2; 124/180; *p < *0.001) and CL flocks: 60.4% (95% CI: 52.3, 68.0; 87/144; *p = *0.002) than in those inoculated with *E. cecorum* from other poultry types – BB: 45.8% (95% CI: 37.9, 54.0; 66/144), T: 41.7% (95% CI: 32.8, 51.1; 45/108), and W: 39.8% (95% CI: 31.1, 49.2; 43/108) (Fig. 1A). The cumulative EMR was significantly higher (*p < *0.001) after infection with clinical chicken strains (CB, BB, CL): 59.2% (95% CI: 54.7, 63.5; 277/468) than with other poultry strains (T, W): 40.7% (95% CI: 34.4, 47.4; 88/216) and commensal strains: 38.9% (Fig. 1C). The comparison of cumulative EMR between *E. cecorum* strains (χ2 test) within the same poultry group showed significant differences for BB, T, and W (*p < *0.001), and no differences for CB (*p = *0.34), CL (*p = *0.057), and commensal (*p = *0.972) groups.

**Table 2:** Summary of embryo mortality by day caused by clinical (CB, BB, CL, T, W) and commensal *Enterococcus cecorum* strains. Results are presented as % mortality (with 95% CI) and the number of deaths / number of surviving embryos. Cumulative EMR is showed as % mortality (with 95% CI) and the number of deaths / total number of inoculated embryos.

Dpi	*E. cecorum* strains derived from different poultry						
	CB (n = 5)	BB (n = 4)	CL (n = 4)	T (n = 3)	W (n = 3)	Commensal (n = 4)	χ2 p-value
1	25 (19, 32) 45/180	33 (26, 41) 47/144	32 (25, 40) 46/144	28 (20, 37) 30/108	26 (19, 35) 28/108	17*↓ (12, 24) 24/144	0.030
2	47*↑ (39, 55) 63/135	17 (10, 25) 16/97	36*↑ (27, 46) 35/98	14 (8, 24) 11/78	11 (6, 20) 9/80	12 (7, 19) 14/120	< 0.001
3	11.1 (6, 20) 8/72	1 (0, 7) 1/81	6 (3, 15) 4/63	5 (2, 12) 3/67	6 (2, 14) 4/71	9 (5, 15) 9/106	0.178
4	7.8 (3, 17) 5/64	1 (0, 7) 1/80	2 (0, 9) 1/59	2 (0, 8) 1/64	2 (0, 8) 1/67	5 (2, 12) 5/97	0.153
5	2 (0, 9) 1/59	0 (0, 5) 0/79	0 (0, 6.2) 0/58	0 (0, 6) 0/63	0 (0, 6) 0/66	3 (1, 9) 3/92	0.154
6	2 (0, 9) 1/58	0 (0, 5) 0/79	0 (0, 6.2) 0/58	0 (0, 6) 0/63	0 (0, 6) 0/66	0 (0, 4) 0/89	0.303
7	2 (0, 9) 1/57	1 (0, 7) 1/79	2 (0, 9) 1/58	0 (0, 6) 0/63	2 (0, 8) 1/66	1 (0, 6) 1/89	0.954
Cumulative EMR	69*↑ (62, 75) 124/180	46 (38, 54) 66/144	60*↑ (52, 68) 87/144	42 (33, 51) 45/108	40 (31, 49) 43/108	39 (31, 47) 56/144	< 0.05

Dpi – days post-infection. EMR – embryo mortality rate.

P-values apply to the comparison of groups indicated by asterisk (*) with other groups using a chi-square test.

Asterisks (*) show statistically significant differences between values in the row.

Arrow shows that the value is significantly higher (↑) or lower (↓).

Table 2 shows the summary of embryo mortality by day and cumulative mortality. Daily EMR was significantly lower in commensal strains than in other groups on day 1 (*p = *0.030) and it was higher in CB and CL strains than in other groups on day 2 (*p < *0.001). EMR was higher in all clinical strains than in commensal strains on day 1 (*p = *0.003) and 2 (*p < *0.001) (Supplementary Table 2), but it was lower on day 5 (*p = *0.009). EMR was lower (*p = *0.010) in commensal strains than in chicken and other poultry strains on day 1. EMR was higher in chicken strains than in other poultry strains and commensal strains on day 2 (*p < *0.001) (Supplementary Table 3). EMR differed (*p < *0.001) between days (from 1 to 7 dpi) within each group of *E. cecorum* strains (in columns: Table 2, Supplementary Table 2, Supplementary Table 3). No mortality was observed in the control group.

### Embryo survival

**Figure 2:**
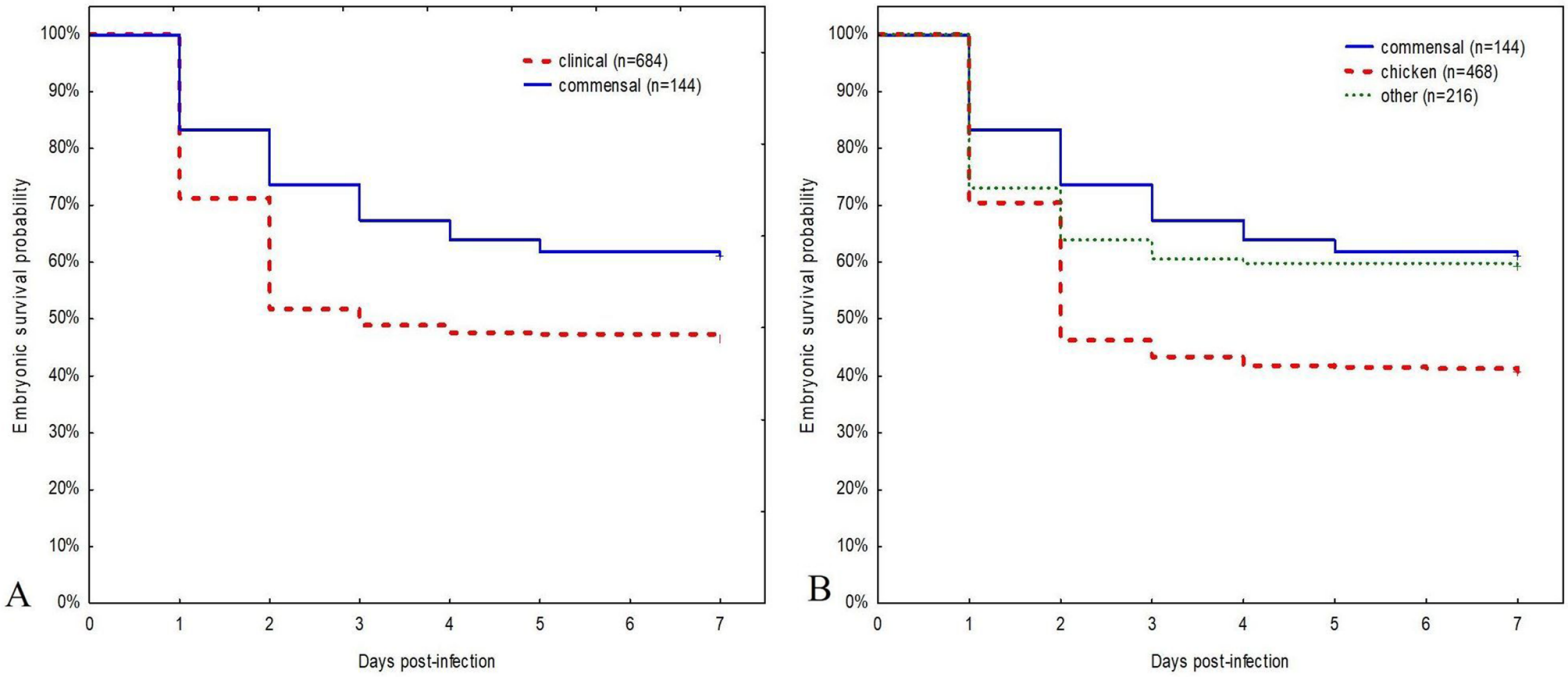
Survival analysis of SPF chicken embryos inoculated with *Enterococcus cecorum*. **(A)** survival comparison between clinical and commensal *E. cecorum* strains **(B)** survival comparison between clinical chicken isolates (CB, BB, CL), clinical non-chicken isolates (T, W) and commensal *E. cecorum* isolates.

Survival rate on 7 dpi was significantly lower in embryos infected with clinical strains (46.6%) than in those infected with commensal (61.1%) strains of *E. cecorum* (log-rank test *p = *0.001) (Fig. 2A). Among clinical *E. cecorum* strains, survival rate on 7 dpi was the lowest in CB (31.1%), followed by CL (39.6%), BB (54.2%), T (58.3%), and W (60.2%) strains. A total of 46.6% embryos survived after infection with clinical strains, 41.4% after infection with broiler (CB, BB) strains, and 40.8% after infection with chicken (CB, BB, CL) strains. Survival rate on 7 dpi was significantly lower in embryos infected with chicken strains (CB, BB, CL) (40.8%) than in embryos infected with other poultry (T, W) strains (59.3%) and commensal strains (61.1%) (Generalized log-rank test *p < *0.001) (Fig. 2B).

### LD_50_

The LD_50_ for *E. cecorum* ranged from 10^2^ to 10^5^ cfu/ml (Table 3) and was the lowest in CB isolates. Compared with commensal isolates, LD_50_ was approx. 800-fold lower in CB isolates, 130-fold lower in chicken isolates (CB, BB, CL), and 34-fold lower in all clinical *E. cecorum* isolates.

**Table 3:** The LD_50_ of clinical (CB, CL, BB, T, W) and commensal *Enterococcus cecorum* strains for SPF chicken embryos.

*E. cecorum* strains derived from different poultry groups	LD_50_ (cfu/ml)
CB	6.07 × 10^2^
CL	2.48 × 10^3^
Chicken (CB, BB, CL)	3.70 × 10^3^
All clinical (CB, BB, CL, T, W)	1.42 × 10^4^
BB	7.30 × 10^4^
T	1.76 × 10^5^
W	3.40 × 10^5^
Commensal	4.80 × 10^5^

### Pathomorphological Lesions

**Figure 3:**
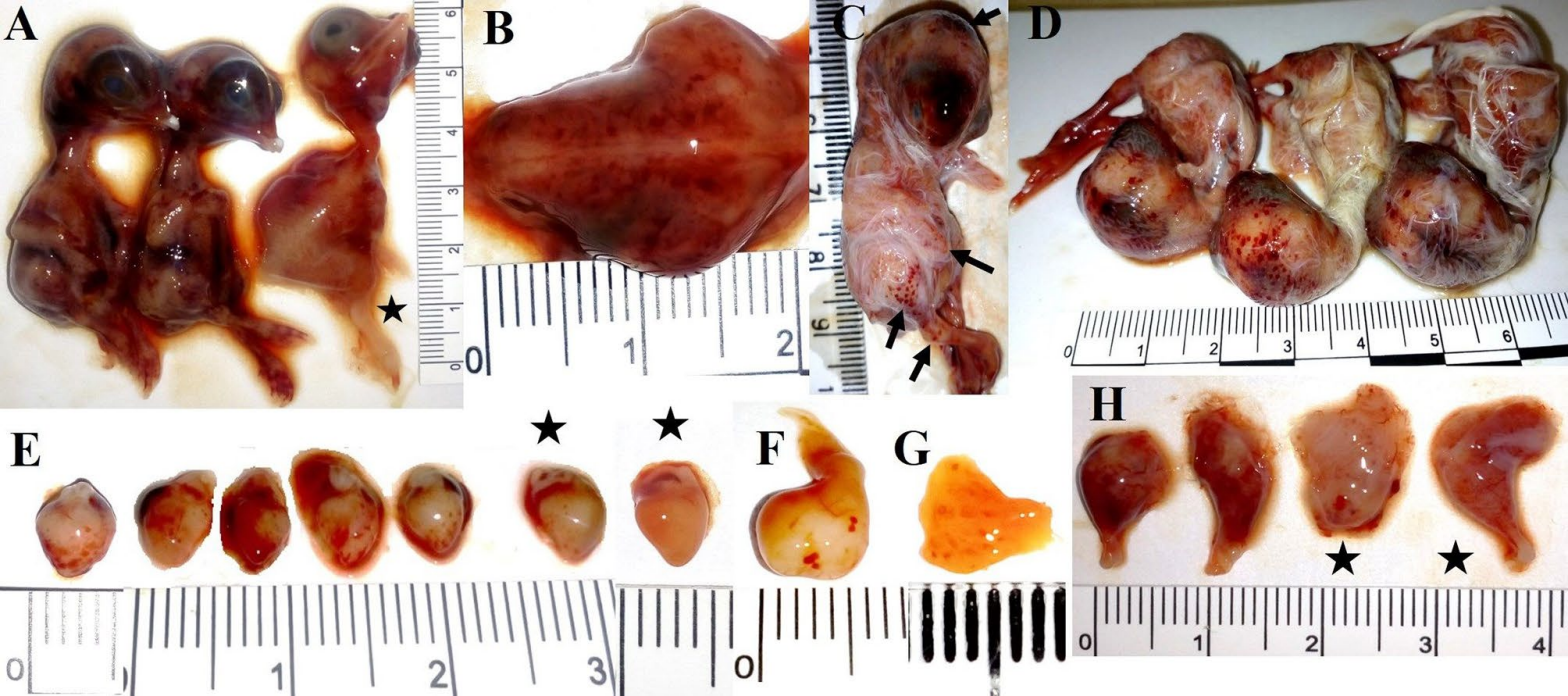
Pathomorphological lesions of SPF chicken embryos (1–4 dpi) inoculated with *Enterococcus cecorum* strains. **(A)** Comparison between congested embryos (2 dpi) infected with clinical *E. cecorum* and embryo infected with commensal (*) *E. cecorum* (approx. 3.4 × 10^6^ cfu/egg) (**B)** Hemorrhages of skeletal muscles in embryos infected with clinical *E. cecorum.* **(C)** Hemorrhages on the head and petechiae on the legs (4 dpi) **(D)** Petechiae on the skull of embryos infected with clinical *E. cecorum*. **(E)** Petechiae on the epicardium of embryos infected with clinical and commensal (*) *E. cecorum* isolates. **(F)** Petechiae on the gizzard (3 dpi) and **(G)** on the mucosa of proventriculus (7 dpi) of embryo infected with clinical *E. cecorum*. **(H)** Hemorrhages on thigh muscles in embryos (2 dpi) infected with clinical (CB, CL, respectively) and commensal (*) *E. cecorum* isolates (approx. 3.4 × 10^7^ cfu/egg).

The most prominent macroscopic lesions occurred in embryos infected with the highest doses (approx. 3.4 × 10^7^ cfu/egg, 3.4 × 10^6^ cfu/egg) of clinical *E. cecorum* that died on 13 di (2 dpi). No lesions were observed in the embryos of the control group. Embryos that died until 15 di (4 dpi) inclusive after infection with chicken isolates (CB, BB, CL) showed severe macroscopic lesions: generalized body congestion, multifocal hemorrhages in the pectoral and thigh muscles, and petechial hemorrhages beneath the epicardium, in the gizzard wall, and in the proventriculus mucosa (Fig. 3). In addition, an abnormal yolk sac content (cloudy, dense, greenish) was noted. The liver was enlarged, friable, congested or greenish in color. The kidneys were congested, the spleen was enlarged, congested or pale, the bursa of Fabricius was enlarged. Embryos infected with commensal isolates showed no or less pronounced lesions. In addition, the surviving embryos (on 7 dpi, 18 di) showed no lesions, but single embryos (receiving the high dose) had abnormal spleen, liver, or yolk sac.

**Figure 4:**
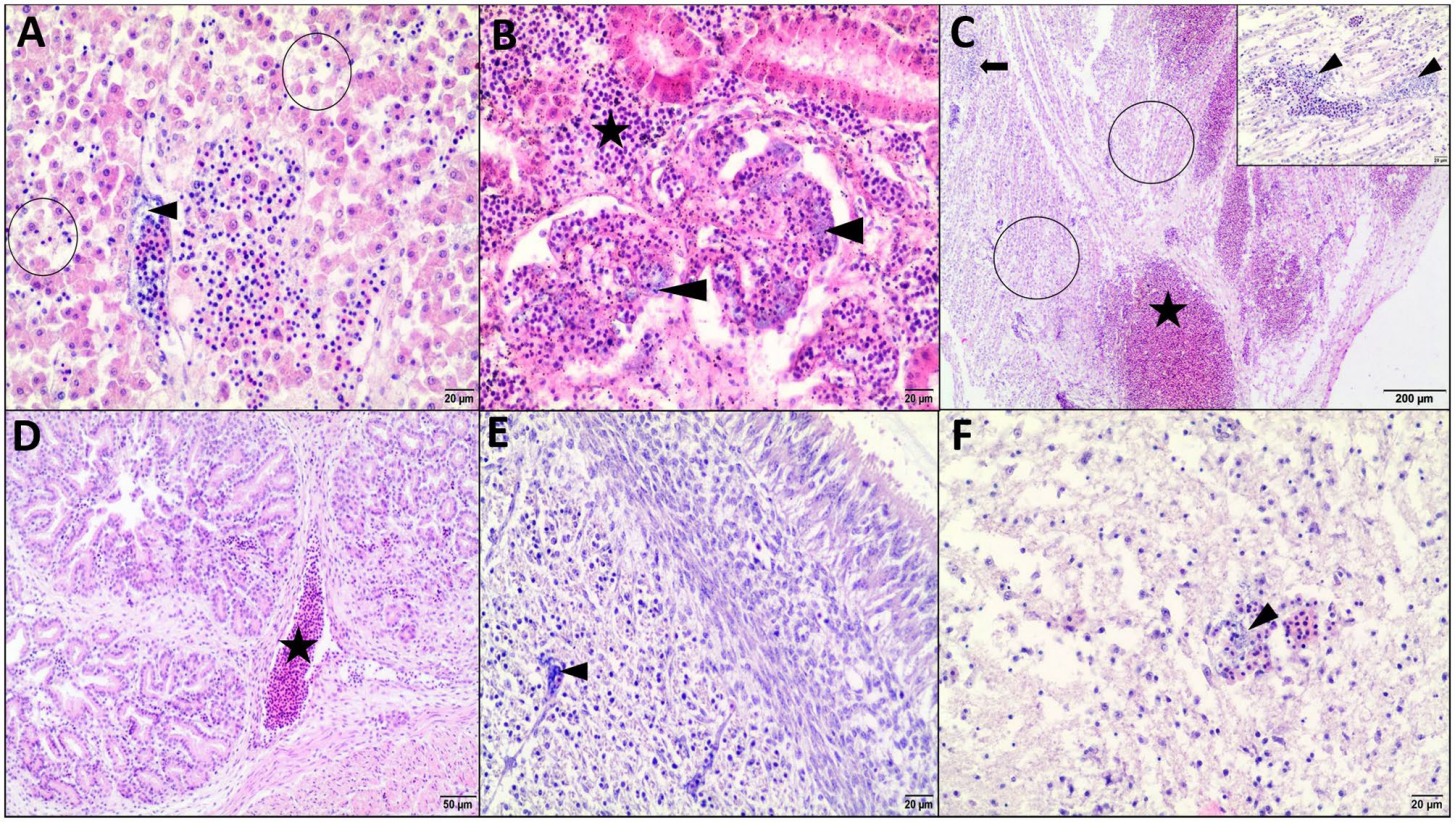
Histopathological lesions in chicken embryos that died (on 13 di, 2 dpi) following infection with clinical *Enterococcus cecorum* strains. HE stain. **(A)** Liver: severe congestion, degenerated and necrotic hepatocytes (circles), loosely arranged hepatic trabeculae, bacterial colonies in blood vessels indicated by arrowhead. Bar: 20 µm. **(B)** Kidneys: severe congestion, hemorrhages (*), capillary blood vessels of glomeruli with bacteria (arrowheads), degenerated and necrotic epithelial cells of renal tubules. Bar: 20 µm. **(C)** Heart: multifocal necrotic areas (circles), massive hemorrhages (*) and severe congestion. Bar: 200 µm; Insert: blood vessels with bacterial aggregates, necrotic cardiomyocytes. Bar: 20 µm. **(D)** Proventriculus. severe congestion (*) and edema of the stroma. Bar: 50 µm. **(E)** Gizzard: diffuse necrotic muscle fibers. Arrowhead indicated blood vessels with bacteria in the muscle layer. Bar: 20 µm. **(F)** Brain: bacterial aggregates (arrowhead) in the blood capillary, pyknosis of glial nuclei and cerebral malacia. Bar: 20 µm.

**Figure 5:**
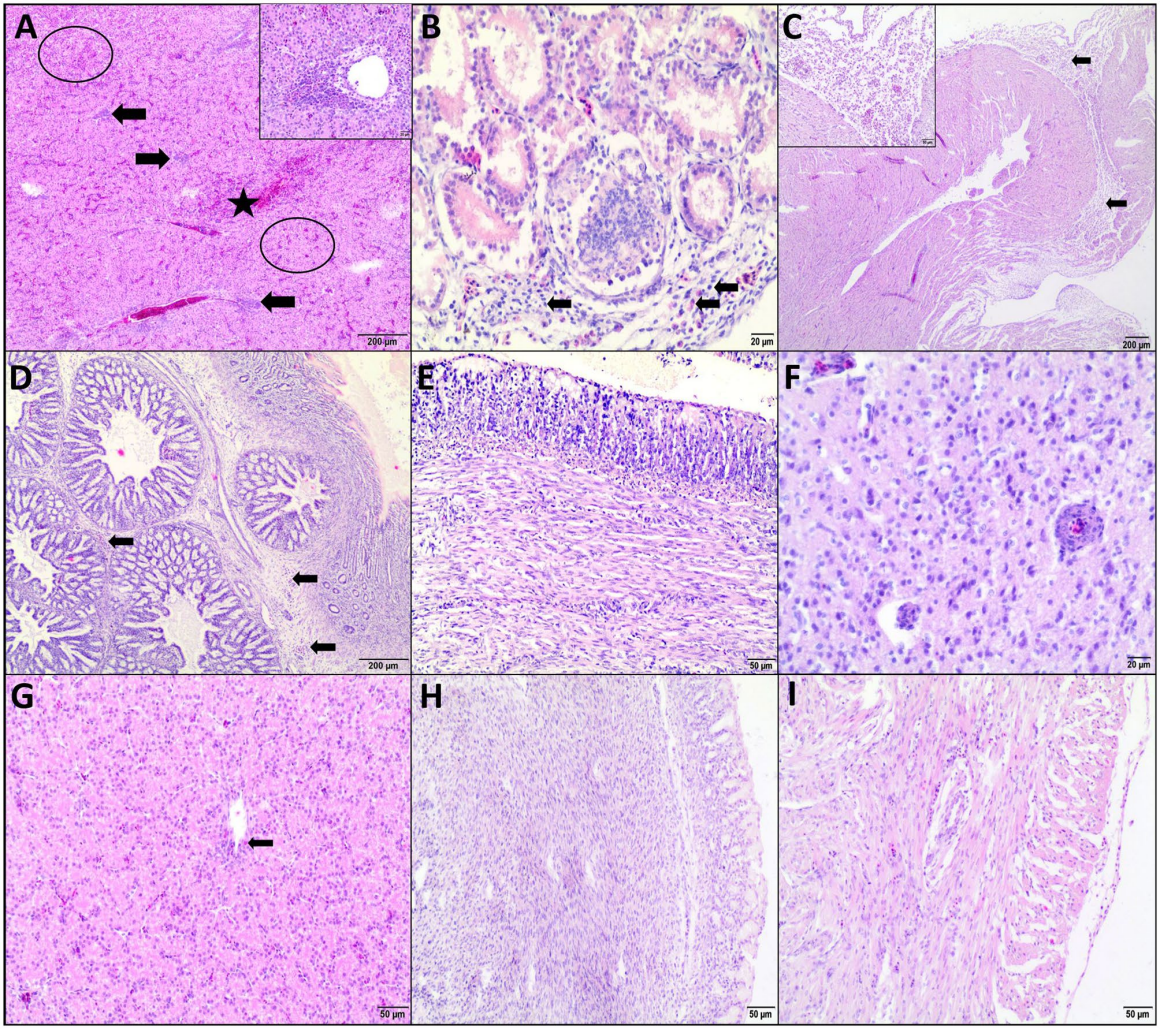
Histopathological lesions in chicken embryos that survived (7 dpi, 18 di) infection with clinical **(A–F)** and commensal **(G–H)** *Enterococcus cecorum* isolates and in non-infected embryos of the control group **(I). (A)** Liver: multifocal areas infiltration of heterophils and mononuclear cells (arrows), necrotic areas (circle), moderate congestion (*). Bar: 200 µm. Insert: moderate degree of inflammatory infiltrate, mild vacuolization of hepatocytes and increased number of bile pigment-laden macrophages. Bar: 20 µm. **(B)** Kidneys: diffuse markedly enlarged glomeruli, vacuolization and necrosis of epithelial cells. Arrows indicated inflammatory cells (heterophils and lymphocytes). Bar: 20 µm. **(C)** Heart: diffuse infiltration of pericardium and epicardium with heterophils and mononuclear cells. Bar: 200 µm. Insert: Bar: 50 µm. **(D)** Proventriculus: mild infiltration of inflammatory cells (heterophils and mononuclear cells) in the tunica intima and between deep glands, edema of the stroma. Bar: 200 µm. **(E)** Gizzard: there was no evidence of inflammation. Bar: 50 µm. **(F)** Brain: severe gliosis, capillary hyperplasia and endothelial hypertrophy, moderate neuropil vacuolization. Bar: 20 µm. **(G)** Liver: mild vacuolization of hepatocytes and infiltration of heterophils and mononuclear cells (arrow) around blood vessels. Bar: 50 µm. **(H)** Gizzard. No presence of inflammation. Bar: 50 µm. **(I)** Heart: no presence of inflammation. Bar: 50 µm.

Histopathological examination of the tissue samples of dead embryos showed aggregates of bacteria in blood vessels in the liver, kidney, heart, gizzard, and brain, multiple areas of necrosis in the liver and heart, and necrosis of renal tubular epithelial cells and gizzard smooth muscle cells. In addition, dead embryos showed encephalomalacia, hemorrhages in the liver, kidney, and heart, as well as marked congestion of the proventriculus, gizzard, and brain (Fig. 4). Tissue samples from the surviving embryos revealed inflammatory response (hepatitis, glomerulonephritis, pericarditis, and proventiculitis), glial and endothelial cell reaction in the brain, and mainly mild to moderate congestion. Bacteria were not found in blood vessels (Fig. 5A–F). Embryos infected with commensal strains showed a mild degree of histopathological lesions (mainly congestion, hepatocyte degeneration, and mild infiltration consisting of heterophils and mononuclear cells) (Fig. 5G–H). Figure 5I shows a representative image of the control group.

### Reisolation of *E. cecorum* after Infection

Pure cultures of *E. cecorum* were reisolated from inoculated embryos, but none of the control group. *E. cecorum* was reisolated from all organs (yolk sac, heart, gizzard) of dead embryos (on 13 di, 2 dpi) and from all organs except gizzard (16.7%) of surviving embryos (on 18 di, 7 dpi).

The number of reisolated *E. cecorum* from the whole heart (cfu/heart) did not significantly differ between clinical and commensal strains in dead embryos and surviving embryos (Table 4) (it was higher in a non-significant manner in commensals). Bacterial loads in the whole heart of embryos that died (on 2 dpi) after inoculation with approx. 3.4 × 10^7^ cfu/egg of *E. cecorum* reached approx. 3.73 × 10^7^ cfu for clinical strains and approx. 10.5 × 10^7^ cfu for commensal strains. The number of reisolated *E. cecorum* from the whole heart of surviving embryos (on 7 dpi) was markedly lower and reached approx. 6.57 × 10^2^ cfu for clinical strains and 18.3 × 10^2^ cfu for commensal strains.

**Table 4:** Bacterial loads in the whole heart [cfu/heart] of dead (13 di, 2 dpi) and surviving (18 di, 7 dpi) SPF chicken embryos inoculated with clinical or commensal *Enterococcus cecorum* strains (at a dose of approximately 3.4 × 10^7^ cfu/egg).

Dead chicken embryos					
Organ	n	Clinical *E. cecorum* [× 10^6^ cfu/heart] Median, IQR (range)	n	Commensal *E. cecorum* [× 10^6^ cfu/heart] Median (range)	*p*-value
Heart (whole)	15	37.281, 5.719–87.29 (0.688–154.8)	4	105.35 (49.02–129.0)	0.124

Surviving chicken embryos					
Organ	n	Clinical *E. cecorum* [cfu/heart] Median, IQR (range)	n	Commensal *E. cecorum* [cfu/heart] Median (range)	*p*-value
Heart (whole)	15	657.0, 164.97–4455 (9–297,000)	4	1829.97 (157.5–2970)	0.852

di – day of incubation; dpi – days post-infection; IQR – interquartile range.

The comparison of the results of bacterial load (cfu/g) between the organs of dead and surviving embryos is shown in Table 5. Reisolation (cfu/g) was significantly higher in all organs of dead embryos compared with that in surviving embryos for clinical (*p < *0.001) and commensal (*p = *0.029) strains. The difference in bacterial load (cfu/g) between the organs of dead embryos was significant only for commensal *E. cecorum* strains (*p = *0.039) (Supplementary Table 4). Reisolation of *E. cecorum* from surviving embryos was significantly higher in the yolk sac than in the heart (*p < *0.001) or gizzard (*p = *0.015) only for clinical strains (Supplementary Table 5). Bacterial loads in organs did not differ significantly between clinical and commensal strains in embryos that died (on 13 days, 2 dpi) (Supplementary Table 4) and in those that survived infection (on 18 di, 7 dpi) (Supplementary Table 5).

**Table 5:** Bacterial loads in the organs [cfu/g] of dead (13 di, 2 dpi) and surviving (18 di, 7 dpi) SPF chicken embryos inoculated with clinical or commensal *Enterococcus cecorum* strains (at dose approx. 3.4 × 10^7^ cfu/egg).

Chicken embryos inoculated with clinical strains of *E. cecorum*			
Organ	Dead embryos (n = 15) Median, IQR (range) [× 10^6^ cfu/g]	Surviving embryos (n = 20) Median, IQR (range) [× 10^6^ cfu/g]	*p*-value
Yolk sac	490, 300–700 (6.66–1400)	0.2033, 0.083–0.7665 (0.0014–183.3)	< 0.001
Heart	867, 133–2030 (16–3600)	0.0073, 0.0018–0.0495 (0.0001–3.3)	< 0.001
Gizzard	533, 197–733 (3.3–2330)	0.0139, 0.0009–0.115 (0–2.83)	< 0.001

Chicken embryos inoculated with commensal strains of *E. cecorum*			
Organ	Dead embryos (n = 4) Median (range) [× 10^6^ cfu/g]	Surviving embryos (n = 4) Median (range) [× 10^6^ cfu/g]	*p*-value
Yolk sac	661.5 (240–867)	0.0992 (0.0002–2.87)	0.029
Heart	2450 (1140–3000)	0.0203 (0.0018–0.033)	0.029
Gizzard	428.5 (200–543)	0.0028 (0–0.0033)	0.029

di – day of incubation; dpi – days post-infection; IQR – interquartile range.

### Body mass

**Figure 6:**
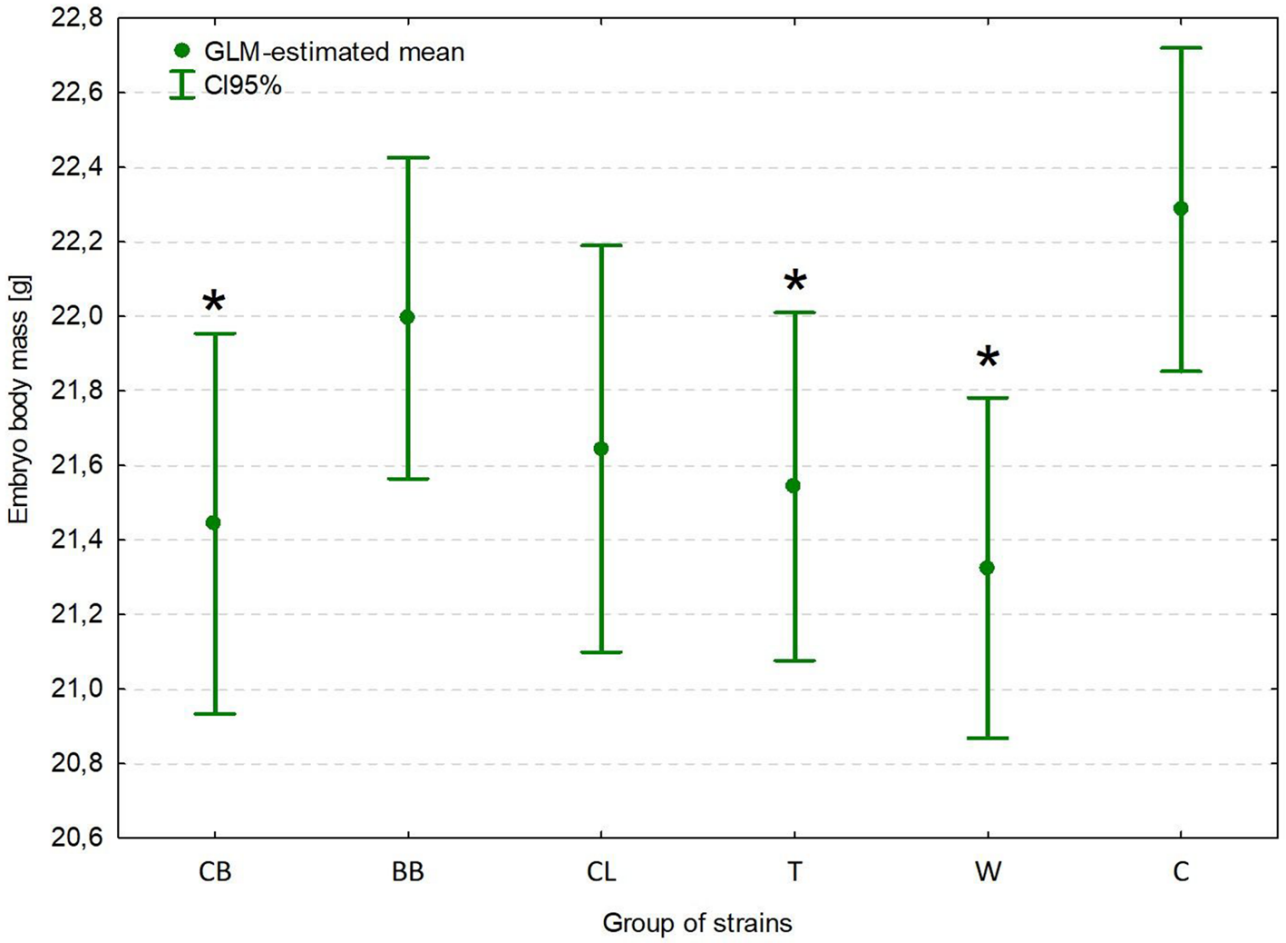
Body mass of SPF chicken embryos (7 dpi, 18 di) inoculated with *Enterococcus cecorum* strains. GLM-estimated mean of the body mass (95% CI).

Results of body mass were presented without the yolk sac contents (yolk-free embryo weight). The inoculating dose did not influence the body mass of embryos. Body mass was significantly lower (*p = *0.006) in embryos infected with clinical strains (21.61 g; 95% CI: 21.39 g, 21.82 g) compared with those infected with commensal strains (22.29 g; 95% CI: 21.85 g, 22.72 g) (Supplementary Table 6). Group of strains (CB, BB, CL, T, W, commensal) had a significant impact (*p = *0.027) on the body mass of surviving embryos. Body mass was significantly lower in embryos infected with clinical strains from CB (21.44 g; 95% CI: 20.93 g, 21.95 g; *p = *0.013), T (21.54 g; 95% CI: 21.08 g, 22.01 g; *p = *0.022) , and W (21.33 g; 95% CI: 20.87 g, 21.78 g; *p = *0.003) compared with those infected with commensal strains (22.29 g; 95% CI: 21.85 g, 22.72 g) (Fig. 6).

## Discussion

The current study investigated the effect of infection of SPF chicken embryos with clinical *E. cecorum* isolates from five different poultry groups (CB, BB, CL, T, W), as well as with commensal *E. cecorum* (C), on embryo survival, pathomorphological lesions, body mass of surviving embryos, and bacterial growth kinetics in embryos.

ELA has demonstrated the ability to discriminate between virulent and avirulent avian *Escherichia coli* and *Rimerella anatipestifer* isolates^36,37^. Previous reports have suggested that ELA may also be a useful method in distinguishing pathogenic and commensal *E. cecorum* strains^25^. Jung et al. observed higher mortality caused by poultry pathogenic *E. cecorum* strains than by commensal strains (approx. 40% vs. 20%)^13^. Furthermore, analysis of *E. cecorum* isolates from various species (chicken, ducks, goose, turkeys, pigeons, budgerigar, swan, cattle, swine, human) showed higher mean total embryo lethality caused by pathogenic strains compared with commensal strains (39.7% vs. 18.9%)^13^. Considering that the cumulative EMR caused by poultry clinical *E. cecorum* strains in our study was significantly higher than that caused by commensal strains (53.4% vs. 38.9%), and EMR caused by chicken strains (CB, BB, CL) was significantly higher than that caused by commensal strains (59.2% vs. 38.9%), our findings support the previous reports. But in fact, clinical *E. cecorum* strains from CB and CL were primarily responsible for this high mortality. According to our best knowledge there is no data concerning the pathogenicity of clinical *E. cecorum* from BB and CL. The only one report concerned commensal strains from CL, and EMR was much lower (approx. 17%) than that provided for clinical CL in our study (60.4%)^13^.

Considering that *E. cecorum* infection is one of the most important bacterial diseases for broiler and broiler breeder flocks (meat chickens)^3,4,6,8,12,14^, we were surprised by the relatively low EMR caused by BB strains (6.9% difference between BB and C is actually a 17% increase for BB over the 38.9% obtained for C). Infection with CB and CL strains caused a significantly higher (approximately twofold) overall EMR compared with commensal strains. Infection with strains from the remaining groups of poultry (BB, T, W) did not produce any significant cumulative EMR compared with commensal strains. Other authors reported no statistically significant ELA results of comparisons between a pathogenic *E. cecorum* strain from broiler and a control (phosphate-buffered saline); however, the variable results for some isolates may have arisen from using different sets of eggs^26^. Although T and W strains caused a lower EMR than CB strains, it was higher than that reported for turkey strain and similar to waterfowl strains^13^. This may indicate a lower pathogenicity of T and W strains, but it may also imply some host species specificity of *E. cecorum* strains. The literature data showed differences in susceptibility between broiler and layer embryos and a low mortality of chicken embryos caused by human *E. cecorum*^13,26^. This finding may support the necessity for use of species-specific embryos in ELA.

As expected, mortality decreased significantly along with decreasing infectious dose. The effect of poultry group and dose on mortality was demonstrated by the significantly higher EMR for CB and CL isolates compared with commensal isolates. However, in some poultry groups, injection of a lower dose was not always associated with the parallel reduction in mortality. Similarly, Ekesi et al.^26^ showed that a lower dose (10^5^ cfu/ml) of pathogenic *E. cecorum* produced slightly more lethality than a higher dose (10^6^ cfu/ml), but neither was statistically different from the PBS control. Other authors reported that some *E. faecalis* strains with the lowest dose (cfu/ml) were able to produce high embryo mortality, whereas other *E. faecalis* strains with a higher dose produced low mortality^29^.

In our opinion, there may be a large variation in EMR between isolates, and ELA may show lower discriminative power. The low pathogenicity of distinct bacterial species including *Staphylococcus* spp., *E. cecorum*, *E. coli* isolated from lame broilers with bacterial chondronecrosis with osteomyelitis (BCO) suggested that ELA might not be an effective measure for assessing bacterial virulence with respect to BCO^26^. Our results revealed the uniformity of EMR among *E. cecorum* isolates within CB, CL, and the commensal group. On the other hand, the strain differences within the BB, T, W groups could have influenced the ELA results. The within-group differences may explain the lower EMR in these groups.

Our study demonstrated significant differences in the survival of embryos infected with clinical and commensal *E. cecorum* strains (46.6% vs. 61.1%). Moreover, survival decreased significantly in embryos infected with clinical chicken strains (CB, BB, CL) compared with other poultry (T, W) strains and commensal strains. Similar observations were described previously for broiler isolates^25^. In contrast to the reduced survival of SPF embryos (9%) and non-SPF broiler embryos (23%) infected with pathogenic *E. cecorum* isolates from broilers in the southeastern United States^25^, the survival rates of embryos infected with pathogenic broiler strains from Poland (this study) and Germany^13^ were higher. This may suggest a lower pathogenicity of European isolates. In contrast, the embryo survival was similar for the commensal *E. cecorum* strains from our study and those from southeastern United States^25^.

Early embryo mortality due to *E. cecorum* inoculation has been observed in previous studies^13,25,26^. The phenomenon may allow drawing meaningful conclusions with respect to the relative pathogenicity of strains^38^. In our study, most of the infected embryos died on 1 dpi, and then EMR gradually decreased. The exception are the CB and CL strains for which EMR was even higher on 2 dpi, and then declined. In the literature, the highest embryo mortality on 2 dpi has been recorded for avian pathogenic *E. coli* (APEC) isolates^39,40^ and on 3 and 4 dpi for avian *E. faecalis*^29^. Massive and rapid (within 2 days) mortality has also been noted for avian pathogenic *E. faecalis* in yolk sac-inoculated embryos^41^. It is noteworthy that in our study, *E. cecorum* strains from T failed to cause mortality after 4 dpi, while other clinical as well as commensal strains caused single deaths on 7 dpi. In contrast to our results, commensal *E. cecorum* strains in another study failed to cause mortality after 2 dpi^25^.

There is limited data on the LD_50_ value of *E. cecorum.* According to the only available results, the 10^2^ dose was the lowest one that reliably achieved mortality greater than 50%^25^. Compared with the previous report, our findings provide LD_50_ values for clinical *E. cecorum* of different poultry and commensal strains. Lower LD_50_ values were obtained for clinical chicken strains and all poultry strains than for commensal strains. As lower LD_50_ is indicative of increased toxicity, we conclude that *E. cecorum* from CB (10^2^ cfu/ml) and CL (10^3^ cfu/ml) showed the highest virulence. The LD_50_ of pathogenic CB *E. cecorum* isolates in our study was approximately 90-fold lower than the LD_50_ (6.6 cfu/ml) of a single avian pathogenic *E. faecalis* strain^22^, which may suggest lower pathogenicity of *E. cecorum* than that of *E. faecalis*; however, further research is needed to confirm and expand on this finding. The literature review indicated that the LD_50_ of *E. cecorum* is lower than that of avian *Mycoplasma gallisepticum* and some *Mycoplasma synoviae* strains^38,42^. It has been reported for *Salmonella* Gallinarum that isolates with a Log_10_LD_50_ of ≤ 4.0 should be considered to be virulent^43^. In our study, CB, CL, and all chicken strains (with log_10_LD_50_ 2.8, 3.4, and 3.6, respectively) may meet the above criterion. However, the differences between the LD_50_ values of clinical and commensal *E. cecorum* strains were lower than those reported for virulent and avirulent *S.* Gallinarum^43^.

So far, only a single study has described gross lesions, while none has focused on histopathological lesions induced by *E. cecorum* in embryos^25^. The high initial EMR caused by the CB and CL *E. cecorum* strains coincided with significant pathomorphological lesions in dead embryos within 2 days. The severity of the pathomorphological lesions was found to be dependent not only on dose (bacterial concentration), but also on the isolate type. Embryos inoculated with the highest dose of clinical chicken (CB, BB, CL) *E. cecorum* isolates showed severe gross lesions involving mainly skeletal muscles, heart, yolk sac, stomach, liver, spleen, and kidneys. Besides the congestion of embryos, we found scattered petechial hemorrhages, usually on the skin (head, legs), organs (heart, gizzard), and mucous membranes (proventriculus). Congested dead embryos with prominent cranial and skin hemorrhages have also been recorded upon infection with *E. coli* (APEC) and *Brucella microti*^21,39,40,44^. Similarly, other authors observed ecchymotic hemorrhages and subcutaneous edema typical of sepsis in embryos inoculated with spinal *E. cecorum* isolates from broilers^25^, different *Enterococcus* species (*E. faecalis*, *E. faecium*, *E. gallinarum*) from meat turkeys^24^, and *Mycoplasma* spp*.*^38,45^. Based on the results obtained herein and in a previous study, all pathomorphological lesions can be associated with the generalized infection (bacteraemia). Contrary to the previous report^25^, minor abnormalities could be found in some embryos infected with a high concentration of commensal *E. cecorum* strains. Furthermore, some survivors exhibited pathomorphological lesions in organs; however, they showed neither dwarfism nor curled toes observed in embryos surviving infection with *Mycoplasma lipofaciens*^45^.

In histopathology, the liver, kidneys, heart, and brain were most affected by *E. cecorum* in dead and surviving embryos. Similarly, microscopic lesions in the heart, brain, and liver were found in embryos inoculated by APEC isolates, but most APEC-induced lesions occurred within 4 days. We found that *E. cecorum* could cause encephalomalacia just as APEC^39^. In contrast to *E. cecorum*, *M. lipofaciens* in another study did not affect the hearts of the embryos^45^. Histopathology provided the evidence of infiltration of heterophils and mononuclear cells in many organs of embryos that survived infection with *E. cecorum* (at a dose of approximately 3.4 × 10^7^/egg) until the end of the study (7 dpi, 18 di), which means that *E. cecorum* can induce the host innate immune response by triggering an inflammatory response. Furthermore, reisolation of viable *E. cecorum* cells from these embryos indicated that *E. cecorum* might survive in tissues affected by the inflammatory process.

Our results showed that *E. cecorum* had a wide tissue distribution. High bacterial loads in heart samples may be due to the bacteraemia and special predilection of *E. cecorum* to the heart. Previous findings have indicated that bacteraemia and intestinal colonization due to pathogenic *E. cecorum* within the first 3 weeks of life are crucial to the pathogenesis of enterococcal spondylitis (ES) in broilers^9^. In this study, we showed that *E. cecorum* effectively replicated in chicken embryos. The number of reisolated *E. cecorum* from the whole hearts of dead embryos upon infection with clinical and commensal strains was higher than the number of bacteria inoculated. Moreover, bacterial loads were lower for clinical than for commensal isolates. This finding, together with the LD_50_ values, clearly indicated the pathogenicity of *E. cecorum* clinical strains. Our results revealed that bacterial loads of 6.57 × 10^2^ cfu/heart for clinical *E. cecorum* and 18.3 × 10^2^ cfu/heart for commensal *E. cecorum* were not mortal for some of 18-day-old chicken embryos. Moreover, the bacterial loads in the whole heart of surviving embryos did not exceed the LD_50_ values determined in this study for clinical and commensal strains, respectively. Although the study was terminated on the 18th day of incubation, we suppose that survivors could probably hatch despite the infection, which may suggest the possibility of transovarian transmission. It is worth mentioning that no strain was recovered from the control (non-infected) embryos, indicating that *E. cecorum* had no airborne dispersion in this study.

For the first time, the impact of *E. cecorum* on the body mass of infected embryos was evaluated. We found that the amount of bacteria in the inoculating dose did not influence the body mass of surviving embryos. However, body mass was significantly lower in the surviving embryos infected with clinical strains than in those infected with commensal strains. It implies that the pathogenicity status of *E. cecorum* strains (clinical vs. commensal), as well as classification to the poultry group strains (CB, BB, CL, T, W), have significant impact on the body mass of surviving embryos. Previous reports have indicated that inoculation of avian pathogenic *E. faecalis* via egg albumen or air-chamber may result in the growth depression of birds, whereas egg-dipping does not affect the weights^41^. According to Montgomery et al.^46^, chicks that hatched from *E. coli*-inoculated embryos revealed increased early mortality, decreased weight gains, and prolonged yolk sac absorption. In our study, infection with clinical *E. cecorum* strains from CB resulted in the lowest body mass of surviving embryos, which may adversely affect the hatch process or chick quality and growth performance.

Further research is needed to investigate the *E. cecorum* replication dynamics and rates in hatched chicks in order to gain a better understanding of bacterial physiology during infection.

## Conclusions

In conclusion, the SPF chicken-embryo model used in this study confirmed the virulence of clinical *E. cecorum* from field outbreaks in 5 different poultry groups (CB, BB, CL, T, W) compared with commensal isolates. Our results indicated the highest pathogenicity of clinical *E. cecorum* isolates from CB and CL. Infection with CB and CL *E. cecorum* isolates resulted in the highest embryo mortality, shortest survival, lowest LD_50_ and severe pathomorphological lesions.

The majority of deaths caused by clinical or commensal *E. cecorum* occurred on 1 dpi. The exception are the CB and CL isolates which caused most deaths on 2 dpi. The deaths resulted from multiple organ damage due to generalized infection. Given that lethality subsided after 2 dpi but that all of the embryos were still infected, those embryos were likely to die or were sickly runts. This may be true also for the commensals. Our data indicated that some SPF embryos could survive infection despite the same inoculating dose and isolate type. The embryos that did not die over the course of the 5-day ELA were still infected and heavily colonized even by the commensals.

For the first time, the LD_50_ values, histopathological lesions, impact on the body mass and tissue colonization following *E. cecorum* infection of embryos have been evaluated. This study provides the first results on the multiplication of *E. cecorum* in chicken embryos. The body mass of surviving embryos is isolate type- rather than dose-dependent. Furthermore, surviving embryos are able to elicit innate immune response to *E. cecorum* infection. At the same time, reisolation of viable bacteria from surviving embryos may suggest that *E. cecorum* evade or resist immune mechanisms in order to successfully persist in organs.

Although ELA revealed significant differences between commensal and clinical (all clinical, all chicken, CB, CL) *E. cecorum* isolates, we suggest that it may not be a valid pathogenicity assay because of the possible variability in some clinical isolates around the triggering of a cytokine cascade in the immature embryo that results in death. However, experimental studies are needed to compare the ability of disease induction by isolates from different poultry groups with high and low EMR. Further studies are also needed to analyse the early host immune response to *E. cecorum* and to determine the correlations between isolate properties and results in ELA.

## Supplementary Information

Supplementary Information.

## Data Availability

The data generated and analysed in this study are included in this published article (and its Supplementary Information files). Other datasets are available from the corresponding author on a reasonable request.
