# Supplementary material for: Chicken embryo lethality assay for determining the lethal dose, tissue distribution and pathogenicity of clinical Enterococcus cecorum isolates from poultry
Source: Sci Rep. 2022 Jun 23;12:10675. doi: 10.1038/s41598-022-14900-9 (PMC9225985; doi:10.1038/s41598-022-14900-9)
Supplement: Supplementary file 1 — Supplementary Information. [file 41598_2022_14900_MOESM1_ESM.docx]

**Chicken embryo lethality assay for determining the lethal dose, tissue distribution and pathogenicity of clinical *Enterococcus cecorum* isolates from poultry**

Beata Dolka^1,^*, Michał Czopowicz^2^, Izabella Dolka^1^, Piotr Szeleszczuk^1^

^1^Department of Pathology and Veterinary Diagnostics, Institute of Veterinary Medicine, Warsaw University of Life Sciences – SGGW, Nowoursynowska 159c St., 02-776 Warsaw, Poland

^2^Division of Veterinary Epidemiology and Economics, Institute of Veterinary Medicine, Warsaw University of Life Sciences – SGGW, Nowoursynowska 159c St., 02-776 Warsaw, Poland

*Corresponding author: beata_dolka@sggw.edu.pl

phone: +48 22 5936035 Department of Pathology and Veterinary Diagnostics, Institute of Veterinary Medicine, Warsaw University of Life Sciences – SGGW, Nowoursynowska 159c St., 02-776 Warsaw, Poland

**Supplementary Table 1.** List of clinical *Enterococcus cecorum* strains included in this study.

| **Strain ID** | **Year of isolation**^12^ | **PFGE pulsotype**^12^ |
| --- | --- | --- |
| CB-2 | 2011 | D |
| CB-22 | 2012 | F |
| CB-31 | 2013 | B |
| CB-95 | 2014 | J |
| CB-150 | 2017 | nd |
| BB-18 | 2012 | B |
| BB-71 | 2014 | E |
| BB-116 | 2013 | H |
| BB-145 | 2016 | C |
| CL-4 | 2011 | C |
| CL-10 | 2011 | B |
| CL-111 | 2013 | nt |
| CL-114 | 2013 | A |
| T-74 | 2014 | nt |
| T-123 | 2016 | nt |
| T-127 | 2016 | A |
| W-29 | 2013 | nt |
| W-63 | 2014 | nt |
| W-104 | 2015 | nt |

nt – denotes that the indicated isolate did not fall into one of the known pulsotypes (non-typeable); nd – no data

^12^ Dolka et al. PLoS ONE 12(9): e0185199. https://doi.org/10.1371/journal.pone.0185199

**Supplementary Table 2.** Summary of embryo mortality by day caused by all clinical and commensal *Enterococcus cecorum* strains.
Results are presented as % mortality (with 95% CI) and the number of deaths / number of surviving embryos. Cumulative EMR is shown as % mortality (with 95% CI) and the number of deaths / total number of inoculated embryos.

| **Dpi** | **Clinical *E. cecorum* strains (n=19)** | **Commensal *E. cecorum* strains (n=4)** | **χ2 p-value** |
| --- | --- | --- | --- |
| 1 | 28.7*↑(25.4, 32.2)  196/684 | 16.7 (11.5, 23.6)  24/144 | 0.003 |
| 2 | 27.5*↑ (23.7, 31.6)  134/488 | 11.7 (7.1, 18.6)  14/120 | <0.001 |
| 3 | 5.6 (3.7, 8.6)  20/354 | 8.5 (4.5, 15.4)  9/106 | 0.280 |
| 4 | 2.7 (1.4, 5.0)  9/334 | 5.2 (2.2, 11.5)  5/97 | 0.222 |
| 5 | 0.3*↓ (0.1, 1.7)  1/325 | 3.3 (1.1, 9.2)  3/92 | 0.009 |
| 6 | 0.3 (0.1, 1.7)  1/324 | 0 (0, 4.1)  0/89 | 0.605 |
| 7 | 1.2 (0.5, 3.1)  4/323 | - 1. (0.2, 6.1)   2. 1/89 | 0.938 |
| Cumulative EMR | 53*↑ (50, 57)  365/684 | 39 (31, 47) 56/144 | 0.002 |

Dpi – day post-infection. EMR – embryo mortality rate.

P-values apply to the comparison of the groups indicated by asterisk (*) with the other groups using a chi-square test.

Asterisks (*) show statistically significant differences between values in the row.
Arrow shows that the value is significantly higher (↑) or lower (↓).

**Supplementary Table 3.** Summary of embryo mortality by day caused by clinical chicken (CB, BB, CL), other poultry (T, W) and commensal *Enterococcus cecorum* isolates. Results are presented as % mortality (with 95% CI) and the number of deaths / number of surviving embryos. Cumulative EMR is shown as % mortality (with 95% CI) and the number of deaths / total number of inoculated embryos.

| **Dpi** | **Clinical chicken (CB, BB, CL) *E. cecorum* strains (n=13)** | **Clinical non-chicken (T, W)  *E. cecorum* strains (n=6)** | **Commensal *E. cecorum* strains (n=4)** | **χ2 p-value** |
| --- | --- | --- | --- | --- |
| 1 | 29.5*↑ (25.5, 33.8)  138/468 | 26.9*↑ (21.4, 33.1)  58/216 | 16.7 (11.5, 23.6)  24/144 | 0.010 |
| 2 | 34.5*↑ (29.6, 39.8)  114/330 | 12.7 (8.3, 18.7)  20/158 | 11.7 (7.1, 18.6)  14/120 | <0.001 |
| 3 | 6.0 (3.6, 10)  13/216 | 5.1 (2.5, 10.1)  7/138 | 8.5 (4.5, 15.4)  9/106 | 0.537 |
| 4 | 3.4 (1.7, 6.9)  7/203 | 1.5 (0.4, 5.4)  2/131 | 5.2 (2.2, 11.5)  5/97 | 0.304 |
| 5 | 0.5 (0.1, 2.8)  1/196 | 0 (0, 2.9)  0/129 | 3.3 (1.1, 9.2)  3/92 | 0.999 |
| 6 | 0.5 (0.1, 2.8)  1/195 | 0 (0, 2.9)  0/129 | 0 (0, 4.1)  0/89 | 0.999 |
| 7 | 1.5 (0.5, 4.4)  3/194 | 0.8 (0.1, 4.3)  1/129 | - 1. (0.2, 6.1)   2. 1/89 | 0.822 |
| Cumulative  EMR | 59*↑ (55, 64) 277/468 | 41 (34, 47) 88/216 | 39 (31, 47) 56/144 | <0.001 |

Dpi – day post-infection. EMR – embryo mortality rate.

P-values apply to the comparison of the groups indicated by asterisk (*) with the other groups using a chi-square test.
Asterisks (*) show statistically significant differences between values in the row.
Arrow shows that the value is significantly higher (↑) or lower (↓).

**Supplementary Table 4.** Comparison of bacterial loads [×10^6^ cfu/g] between organs and between types of *Enterococcus cecorum* isolates (clinical vs. commensal) in SPF chicken embryos that died (13 di, 2 dpi) after inoculation with *E. cecorum* (approx. 3.4×10^7^ cfu/egg).

| **Embryos that died**  **Median, IQR (range) [×10^8^ cfu/g]** | | | | |
| --- | --- | --- | --- | --- |
| **Type of *E. cecorum*** | **Yolk sac** | **Heart** | **Gizzard** | **p-value** |
| Clinical (n=15) | 4.90,  3.00–7.00 (0.07–14.00) | 8.67,  1.33–20.30 (0.16–36.00) | 5.33,  1.97–7.33 (0.03–23.30) | Friedmann test p=0.247 |
| Commensal (n=4) | 6.62,  4.32–7.84 (2.40–8.67) | 24.50,  16.85–28.35 (11.40–30.00) | 4.29,  3.00–5.00 (2.00–5.43) | Friedmann test p=0.039  Wilcoxon with Bonferroni correction  1 vs. 2 p=0.204  2 vs. 3 p=0.204  1 vs. 3 p=0.819 |
| Mann–Whitney U test | p=0.423 | p=0.121 | p=0.617 |  |

IQR – interquartile range

**Supplementary Table 5.** Comparison of bacterial loads [×10^3^ cfu/g] between organs and types of *Enterococcus cecorum* isolates (clinical vs. commensal) in SPF chicken embryos that survived until the end of the study (18 di, 7 dpi) after inoculation with *E. cecorum* (approx. 3.4×10^7^ cfu/egg).

| **Embryos that survived  Median, IQR (range) [×10^3^ cfu/g]** | | | | |
| --- | --- | --- | --- | --- |
| **Type of *E. cecorum*** | **Yolk sac** | **Heart** | **Gizzard** | **p-value** |
| Clinical (n=20) | 203.3,  83.00–766.50 (1.43–183300) | 7.30,  1.83–49.50 (0.10 –3300) | 13.90,  0.92–115.00 (0–2830) | Friedmann test p<0.001  Wilcoxon with Bonferroni correction  1 vs. 2 p<0.001  2 vs. 3 p=0.999  1 vs. 3 p=0.015 |
| Commensal (n=4) | 99.15,  29.27–1505 (0.23–2870) | 20.33,  4.71–33.00 (1.75–33.00) | 2.75,  1.10–3.30 (0–3.30) | Friedmann test p=0.039  Wilcoxon with Bonferroni correction  1 vs. 2 p=0.432  2 vs. 3 p=0.204  1 vs. 3 p=0.204 |
| Mann–Whitney U test | p=0.373 | p=0.846 | p=0.187 |  |

IQR – interquartile range

**Supplementary Table 6.** Body mass of chicken embryos that survived until the end of the experiment (18 di, 7 dpi) after inoculation with *Enterococcus cecorum*.

| **Inoculum**  **(cfu/ml)** | **n** | **Clinical *E. cecorum*** | **n** | **Commensal *E. cecorum*** |
| --- | --- | --- | --- | --- |
|  |  | **Body Mass**  **Mean ± SD (range)** |  | **Body Mass**  **Mean ± SD (range)** |
| 3.4 × 10^8^ | 25 | 22.05 ± 2.69 (13.28–25.48) | 7 | 22.88 ± 1.30 (21.30–24.89) |
| 3.4 × 10^7^ | 25 | 21.68 ± 1.53 (16.89–23.86) | 6 | 23.36 ± 0.92 (21.77– 24.27) |
| 3.4 × 10^6^ | 24 | 21.63 ± 1.94 (15.97–24.99) | 8 | 21.62 ± 1.72 (17.45– 22.84) |
| 3.4 × 10^5^ | 26 | 21.39 ± 2.15 (15.75–25.02) | 8 | 22.22 ± 1.55 (19.33–24.96) |
| 3.4 × 10^4^ | 32 | 21.79 ± 1.36 (19.22–24.02) | 7 | 23.53 ± 0.95 (22.64–25.14) |
| 3.4 × 10^3^ | 34 | 21.29 ± 1.85 (15.47–24.67) | 6 | 22.93 ± 1.62 (20.46–24.79) |
| 3.4 × 10^2^ | 35 | 21.54 ± 1.56 (17.71–24.28) | 10 | 22.11 ± 1.82 (19.5–24.02) |
| 3.4 × 10^1^ | 46 | 21.78 ± 1.55 (17.74–25.28) | 12 | 21.53 ± 1.63 (19.23–24.13) |
| 3.4 | 55 | 21.17 ± 1.75 (13.86–23.94) | 8 | 21.34 ± 4.61 (10.46–24.88) |

Dpi – day post-infection. General linear models (GLM): Dilution F_8.364_ = 1.19, p=0.303; Type of strain F_1.364_ = 7.70, p=0.006.
SD – standard deviation
